# Linear discriminant analysis of phenotypic data for classifying autism spectrum disorder by diagnosis and sex

**DOI:** 10.3389/fnins.2022.1040085

**Published:** 2022-11-16

**Authors:** Zachary Jacokes, Allison Jack, Catherine A. W. Sullivan, Elizabeth Aylward, Susan Y. Bookheimer, Mirella Dapretto, Raphael A. Bernier, Daniel H. Geschwind, Denis G. Sukhodolsky, James C. McPartland, Sara J. Webb, Carinna M. Torgerson, Jeffrey Eilbott, Lauren Kenworthy, Kevin A. Pelphrey, John D. Van Horn, The GENDAAR Consortium, Katy Ankenman

**Affiliations:** ^1^Laboratory of Brain and Data Science, Department of Psychology, School of Data Science, University of Virginia, Charlottesville, VA, United States; ^2^Department of Psychology, George Mason University, Fairfax, VA, United States; ^3^Department of Pediatrics, Yale School of Medicine, New Haven, CT, United States; ^4^Department of Psychiatry and Behavioral Sciences, University of Washington, Seattle, WA, United States; ^5^Department of Psychiatry and Biobehavioral Sciences, University of California, Los Angeles, Los Angeles, CA, United States; ^6^Center for Neurobehavioral Genetics, University of California, Los Angeles, Los Angeles, CA, United States; ^7^Child Study Center, Yale School of Medicine, New Haven, CT, United States; ^8^Center on Child Health, Behavior, and Development, Seattle Children’s Research Institute, Seattle, WA, United States; ^9^Neuroscience Graduate Program, University of Southern California, Los Angeles, CA, United States; ^10^Center for Autism Spectrum Disorders, Children’s National Hospital, Washington, DC, United States; ^11^Department of Neurology, University of Virginia, Charlottesville, VA, United States

**Keywords:** autism spectrum disorder, phenotypic analysis, multivariate statistics, classification, diagnostic

## Abstract

Autism Spectrum Disorder (ASD) is a developmental condition characterized by social and communication differences. Recent research suggests ASD affects 1-in-44 children in the United States. ASD is diagnosed more commonly in males, though it is unclear whether this diagnostic disparity is a result of a biological predisposition or limitations in diagnostic tools, or both. One hypothesis centers on the ‘female protective effect,’ which is the theory that females are biologically more resistant to the autism phenotype than males. In this examination, phenotypic data were acquired and combined from four leading research institutions and subjected to multivariate linear discriminant analysis. A linear discriminant model was trained on the training set and then deployed on the test set to predict group membership. Multivariate analyses of variance were performed to confirm the significance of the overall analysis, and individual analyses of variance were performed to confirm the significance of each of the resulting linear discriminant axes. Two discriminant dimensions were identified between the groups: a dimension separating groups by the diagnosis of ASD (LD1: 87% of variance explained); and a dimension reflective of a diagnosis-by-sex interaction (LD2: 11% of variance explained). The strongest discriminant coefficients for the first discriminant axis divided the sample in domains with known differences between ASD and comparison groups, such as social difficulties and restricted repetitive behavior. The discriminant coefficients for the second discriminant axis reveal a more nuanced disparity between boys with ASD and girls with ASD, including executive functioning and high-order behavioral domains as the dominant discriminators. These results indicate that phenotypic differences between males and females with and without ASD are identifiable using parent report measures, which could be utilized to provide additional specificity to the diagnosis of ASD in female patients, potentially leading to more targeted clinical strategies and therapeutic interventions. The study helps to isolate a phenotypic basis for future empirical work on the female protective effect using neuroimaging, EEG, and genomic methodologies.

## Introduction

Autism Spectrum Disorder (ASD) is a developmental disability characterized by social and communication deficits (Elsabbagh and Johnson, 2007). Recent research suggests ASD affects 1 in 44 children in the United States (Christensen et al., 2018); this number has increased in recent years for several possible reasons: screening has improved, prevalence of ASD may in fact be increasing, and diagnostic capabilities may have improved. ASD diagnoses are usually confirmed when a child is quite young, and it is generally understood that earlier diagnoses and interventions result in more favorable social outcomes for those affected by ASD (Itzchak, 2011). Evidence suggests that ASD is diagnosed more commonly in males (Halladay et al., 2015; Irimia et al., 2017), though it is unclear whether this diagnostic disparity is a result of a biological predisposition or limitations in referral patterns, screening devices, and diagnostic tools, or all of these combined. One hypothesis centers around the ‘female protective effect,’ which is the theory that females are biologically more resistant to the autism phenotype than males, to the point where they must be more severely affected to be classified as ASD by our current diagnostic standards (Robinson et al., 2013; Gockley et al., 2015; Zhang et al., 2020). To disambiguate this phenomenon, phenotypic survey batteries have become standard in autism research to better understand what behavioral and other measurable characteristics differentiate neurotypical children from their ASD counterparts. Indeed, previous research suggests that girls require a stronger manifestation of autistic traits to meet diagnostic criteria, which in turn suggests that girls are more likely to have ASD and not be diagnosed than boys (Ratto et al., 2018). Additional research has found that girls with ASD do exhibit a distinct behavioral profile, particularly in terms of ability to adapt behavior based on social context (Hiller et al., 2014), desire to be liked by others (Hiller et al., 2016), and ability to mesh within a same-sex social group (Dean et al., 2014; McQuaid et al., 2021). Aside from proposed biological mechanisms, phenotypic analyses may be able to identify which behavioral domains are most implicated in ASD diagnoses and whether redundancy and inefficiency can be identified within these measurement tools.

The phenotypic measures examined here were carefully selected to effectively capture the behavioral expression of these participants. The phenotype battery includes assessments of intelligence, executive function, language, and social skills (detailed further in the section “Materials and methods”). These domains provide a robust baseline by which we can differentiate behavioral characteristics between ASD and neurotypical participants, and between ASD males and ASD females.

The data discussed in this report is from a multimodal, longitudinal study on ASD uniquely suited to identify the cause of this apparent diagnostic discrepancy. The study consists of neuroimaging, EEG, genomic, and phenotypic data; as an initial assessment, only the phenotypic data is examined here. Eventually a large-scale multimodal analysis will be performed on these data to realize the full potential of this unique dataset, but a preliminary phenotypic analysis should provide a meaningful foundation on which future analyses can build. The following report is the initial attempt at classification analysis comprising all of the subscales of the phenotypic measures used in the study.

## Materials and methods

### Participants

Phenotypic data were acquired across four satellite institutions: (1) the Center for Translational Developmental Neuroscience, Child Study Center, Yale School of Medicine, New Haven, CT (*n* = 85 participants); (2) the Nelson Laboratory of Cognitive Neuroscience, Boston Children’s Hospital, Harvard Medical School, Boston, MA (*n* = 57 participants); (3) the Center on Human Development & Disability, Seattle Children’s Hospital, University of Washington School of Medicine, Seattle, WA (*n* = 125 participants); (4) Staglin IMHRO Center for Cognitive Neuroscience, David Geffen School of Medicine, University of California, Los Angeles, CA (*n* = 113 participants). The study was undertaken in agreement with US federal law (45 CFR 46) and has been approved by the Institutional Review Boards at each of the participating data acquisition sites. Participants were recruited to be within a limited age range (range: 8–18 years old), and the diagnostic and sex ratios were intended to be balanced, including 203 ASD participants (92 female) and 177 typically developing (TD) control participants (85 female) for a total of N = 380 participants (177 female). Informed consent was obtained from all participants and from their legally authorized representatives.

### Inclusion/Exclusion criteria

Diagnosis and inclusion of the ASD participants was based on having a prior clinical or research diagnosis of ASD, the Autism Diagnostic Interview (ADI) and the Autism Diagnostic Observation Schedule (ADOS-3 and 4). For ADI inclusion, participants must have scored: greater than 8 on the communication subtotal; greater than 6 on the behavioral subtotal; greater than 1 on the social affect subtotal; greater than 18 on the sum of the previous three subtotals. For ADOS inclusion, ASD participants must have scored higher than 3 on the comparison score (used to compare across Modules 3 and 4). Both requirements needed to be satisfied for inclusion. For the comparison group, participants had no previously reported autism symptoms via parent report on the Social Reciprocity Scale (*T*-score < 60) or the Social Communication Questionnaire (raw score < 11), as well as no clinical impression of ASD. The comparison group was also devoid of diagnosis or behaviors suggestive of schizophrenia or any other learning, developmental, or psychiatric disorder. We previously report sex differences in developmental milestones and diagnostic variables in this sample of autistic youth (Harrop et al., 2021). All participants were required to score higher than 70 on the Differential Ability Scale composite measure of conceptual ability (an IQ proxy).

Exclusion of ASD participants was based on the presence of non-ASD-related genetic, neurological, or psychiatric comorbidity, including use of benzodiazepine, barbiturate, or anti-epileptic medication. Exclusion for the control participants included diagnosed, referred, or suspected ASD, schizophrenia, learning or intellectual disability, any other developmental or psychiatric disorders, and any first- or second-degree relative with ASD.

### Recruitment and data collection

Participants were screened by reliably trained clinicians by telephone and in-person to ensure inclusion and exclusion criteria were met. The phenotypic measures that required clinician administration were collected in-person; these include: Differential Ability Scales-II (DAS) (Elliott et al., 2018), Vineland Adaptive Behavior Scales-II (VABS) (Sparrow et al., 2012), Clinical Evaluation of Language Fundamentals (CELF) (Semel et al., 2003). Phenotypic measures that were parent-report were completed at home by the family; these include: the Social Responsiveness Scale (SRS) (Constantino, 2013), Repetitive Behaviors Scale – Revised (RBSR) (Bodfish et al., 2014), the Child Behavior Checklist (CBCL) (Achenbach and Rescorla, 2000), and the Behavior Rating Inventory of Executive Function (BRIEF-2) (Gioia et al., 2017). In all, 35 predictors were included in this analysis. A full accounting of the demographic and clinical characteristics of the dataset can be found in Table 1.

**TABLE 1:** Demographic and clinical characteristics of the data.

	ASD female	ASD male	TD female	TD male	Total
Age	151.62 (34.74)	151.54 (35.65)	156.50 (38.22)	159.55 (32.73)	154.61 (35.38)
Age at diagnosis	95.80 (148.38)	84.01 (135.15)			89.16 (140.37)
ADI-R: A Total	18.33 (6.29)	19.04 (5.80)			18.72 (6.02)
ADI-R: Bv Total	15.51 (4.65)	16.07 (4.60)			15.82 (4.62)
ADI-R: C Total	5.86 (2.70)	6.19 (2.70)			6.04 (2.70)
ADI-R: D Total	2.97 (1.18)	3.43 (1.22)			3.22 (1.22)
ADOS-3: Overall Total	9.75 (4.19)	13.88 (3.76)			12.50 (4.23)
BRIEF Emotional Control	62.16 (12.07)	61.63 (11.62)	43.75 (7.68)	44.43 (7.28)	53.50 (13.36)
BRIEF Inhibit	66.13 (14.06)	62.35 (13.18)	43.46 (4.49)	45.43 (7.77)	54.83 (14.63)
BRIEF Initiate	66.03 (11.62)	66.35 (11.40)	45.90 (7.46)	46.42 (9.11)	56.77 (14.22)
BRIEF Monitor	67.91 (11.61)	64.46 (11.49)	43.16 (7.64)	44.36 (8.91)	55.55 (15.09)
BRIEF Organization Materials	58.81 (10.37)	57.01 (11.81)	49.14 (10.26)	49.12 (8.85)	53.73 (11.30)
BRIEF Plan/Organize	68.56 (12.50)	65.05 (10.95)	46.55 (7.94)	45.47 (8.49)	56.92 (14.54)
BRIEF Shift	68.20 (13.16)	69.73 (13.38)	43.43 (6.17)	44.06 (7.83)	57.13 (16.61)
BRIEF Working Memory	68.35 (11.76)	67.15 (11.78)	46.86 (8.32)	46.87 (9.08)	57.89 (14.72)
CBCL Aggressive	59.93 (9.45)	57.96 (8.32)	51.17 (2.51)	51.19 (2.79)	55.25 (7.73)
CBCL Anxious	63.46 (10.43)	59.68 (8.47)	52.69 (4.90)	52.28 (4.69)	57.20 (8.89)
CBCL Attention	68.32 (11.49)	64.35 (9.37)	52.06 (3.49)	51.99 (3.83)	59.51 (10.72)
CBCL Rulebreak	56.94 (6.21)	55.47 (6.24)	51.64 (2.89)	51.13 (2.40)	53.90 (5.44)
CBCL Social Problems	64.88 (9.42)	63.25 (8.37)	51.28 (2.40)	51.59 (4.14)	58.07 (9.29)
CBCL Somatic Complaints	59.69 (8.77)	57.49 (7.05)	53.69 (5.38)	53.39 (6.44)	56.16 (7.48)
CBCL Thought	65.75 (9.43)	64.19 (8.84)	52.37 (4.35)	52.08 (3.91)	58.94 (9.60)
CBCL Withdrawn	64.26 (11.26)	61.91 (8.78)	52.85 (4.25)	52.59 (3.82)	58.15 (9.33)
CELF Formulate Sentences	9.32 (3.78)	8.09 (3.70)	11.66 (1.82)	11.48 (2.69)	9.99 (3.50)
CELF Recall Sentences	8.95 (3.78)	8.00 (3.84)	11.24 (2.77)	10.93 (2.56)	9.64 (3.59)
DAS Special Non-verbal	99.40 (19.52)	101.33 (18.08)	108.81 (14.22)	110.64 (16.30)	104.71 (17.84)
DAS Spatial Reasoning	98.53 (18.45)	100.19 (17.01)	107.72 (13.10)	109.74 (15.56)	103.78 (16.87)
DAS Verbal Reasoning	101.92 (21.13)	101.10 (20.06)	110.89 (15.27)	112.14 (18.79)	106.15 (19.66)
RBS-R Compulsive	3.28 (3.53)	3.10 (4.00)	0.43 (0.87)	0.45 (1.47)	1.89 (3.18)
RBS-R Restricted	2.16 (2.20)	3.41 (2.77)	0.06 (0.29)	0.24 (0.94)	1.58 (2.37)
RBS-R Ritualistic	4.72 (4.44)	4.30 (3.68)	0.29 (0.92)	0.49 (2.00)	2.55 (3.73)
RBS-R Sameness	7.78 (8.22)	7.25 (6.24)	0.57 (1.39)	0.87 (2.93)	4.29 (6.41)
RBS-R Self-Injurious	1.64 (2.07)	2.09 (3.08)	0.12 (0.36)	0.15 (0.50)	1.06 (2.15)
RBS-R Stereotyped	3.20 (3.16)	3.14 (3.37)	0.12 (0.57)	0.22 (1.06)	1.75 (2.87)
SRS Awareness	72.21 (11.22)	69.99 (11.41)	46.65 (8.35)	46.66 (10.89)	59.40 (16.14)
SRS Cognition	73.31 (10.33)	69.69 (10.18)	44.82 (6.00)	45.33 (9.46)	58.80 (16.10)
SRS Communication	75.57 (10.82)	71.74 (10.88)	46.28 (6.83)	45.42 (9.53)	60.29 (16.98)
SRS Motivation	70.42 (13.40)	66.30 (12.43)	48.06 (9.51)	46.92 (9.57)	58.30 (15.47)
SRS Restricted/Repetitive	75.16 (11.85)	71.31 (11.59)	45.99 (5.94)	46.17 (8.38)	60.40 (16.77)
Vineland Communication	77.44 (13.02)	74.36 (9.36)	100.16 (14.29)	95.86 (12.86)	86.04 (16.64)
Vineland Living Skills	78.12 (14.71)	74.56 (11.94)	98.45 (13.87)	94.25 (14.63)	85.46 (17.08)
Vineland Socialization	72.41 (12.53)	72.20 (10.69)	100.91 (13.01)	99.80 (12.47)	85.25 (18.48)

The DAS is designed to assess intellectual functioning in school-aged children across several domains: verbal reasoning, non-verbal reasoning, and spatial reasoning. The Special Non-verbal Composite was used instead of the general non-verbal reasoning standard score because it has been shown to more accurately reflect the wide range of verbal capabilities for those affected with ASD (Riccio et al., 1997; Thurman and Hoyos Alvarez, 2020). For this analysis the standardized scores for each of these domains were used.

The VABS is designed to measure adaptive behavior skills required for day-to-day life. It has been used to help diagnose and classify developmental disorders, notably in those affected by ASD. VABS data was collected by parent report and is analyzed here using the standard scores of the main three domains: Communication, Socialization, and Daily Living Skills.

The BRIEF-2 is a measure designed to assess executive function in children and adolescents. It is comprised of three overarching indices (behavior regulation, emotional regulation, and cognitive regulation) with several domains within each index. The domains included in this analysis are the following: inhibit, monitor, shift, emotional control, initiate, working memory, plan/organize, and organization of materials. The overall indices were not included in order to achieve a more granular phenotypic analysis.

The SRS measures autistic traits across five domains: Social Awareness, Social Cognition, Social Communication, Social Motivation, and Restricted Repetitive Behaviors. This measure was specifically designed to help understand the impairments present in ASD relative to neurotypical individuals.

The CELF is designed to evaluate language and communication skills. It consists of several independent subscales, but unfortunately due to large amounts of missing data in our sample only two have been included in this analysis: recalling sentences and formulating sentences standard scores. These two domains should provide a reasonable representation of language ability (Klem et al., 2015).

The RBSR is a measure designed specifically for use in the ASD population. It measures the quality and quantity of repetitive behavior in children with ASD, which is a fundamental aspect of the ASD phenotype. The six subdomains included in this analysis are the following: stereotyped behavior, self-injurious behavior, compulsive behavior, ritualistic behavior, sameness behavior, and restricted behavior. Raw scores were used since this measure does not provide standard scores.

The CBCL is designed to measure symptoms of emotional and behavioral problems in children and adolescents. The eight narrow-band syndrome scale T-scores were included in this analysis: Anxious Behavior, Withdrawn Behavior, Somatic Complaints, Social Problems, Thought Problems, Attention Problems, Rule Breaking Behavior, and Aggressive Behavior. We had previously reported sex differences in aggression in autistic male and female youth (Neuhaus et al., 2022).

### Statistical analysis

Any measures missing more than 10 percent of the sample data points were removed from this analysis; these include the CELF-Receptive Word Classes, and CELF-Expressive Word Classes. For any remaining missing data, the Predictive Mean Matching data imputation method was implemented since the missing data was assumed to be missing at random. Data imputation was performed in R using the Multivariate Imputation via Chained Equations (MICE) and Visualization and Imputation of Missing Values (VIM) packages.

Participants were classified into four nominal classes: ASD male, ASD female, non-autistic male, and non-autistic female. Participants were randomly split into training and testing groups (75% training, 25% testing), and cross-validation was repeated ten times to ensure balance in the splits. A linear discriminant model was trained on the training set and then deployed on the test set to predict group membership.

Multivariate analyses of variance were performed to confirm the significance of the overall analysis, and individual analyses of variance were performed to confirm the significance of each of the resulting linear discriminant axes. All analyses were performed using the R statistical programming language utilizing the following packages: Classification and Regression Training (caret), and Modern Applied Statistics with S (MASS). The visualization of results was performed using the tidyverse and ggplot2 packages.

## Results

Table 2 shows the resulting confusion matrix from the initial linear discriminant analysis. The overall classification accuracy on the test set was 62.766%. Table 3 shows the classification statistics by class. Sensitivity (true positive rate) for the ASD groups was 65.22% for ASD females and 59.26% for ASD males; sensitivity for the control groups was 57.14% for the control females and 69.57% for the control males. Precision (positive predictive value) for the ASD groups was 55.56% for ASD females and 72.73% for ASD males; precision for the control groups was 57.14% for control males and 66.67% for control females.

**TABLE 2:** Confusion matrix from the linear discriminant analysis on the test data.

		Actual				
		
		ASD female	ASD male	TD female	TD male	Totals
**Predicted**	ASD Female	14	8	1	2	25
	ASD Male	8	19	0	0	27
	TD Female	1	0	9	4	14
	TD Male	0	0	11	17	28
	Totals	23	27	21	23	94

TD, typically developing control.

**TABLE 3:** Individual class statistics from the linear discriminant analysis.

	ASD female (N = 23)	ASD male (N = 27)	TD female (N = 21)	TD male (N = 23)
Sensitivity	60.87%	70.37%	42.86%	73.91%
Specificity	84.51%	88.06%	93.15%	84.51%
Precision	56.00%	70.37%	64.29%	60.71%
Prevalence	24.47%	28.72%	22.34%	24.47%
Balanced Accuracy	72.69%	79.22%	68.00%	79.21%
F-1 Score	58.33%	70.37%	51.43%	66.67%

Sensitivity = true positive rate, specificity = true negative rate, precision = positive predictive value. Overall accuracy = 62.77%.

**FIGURE 1:**
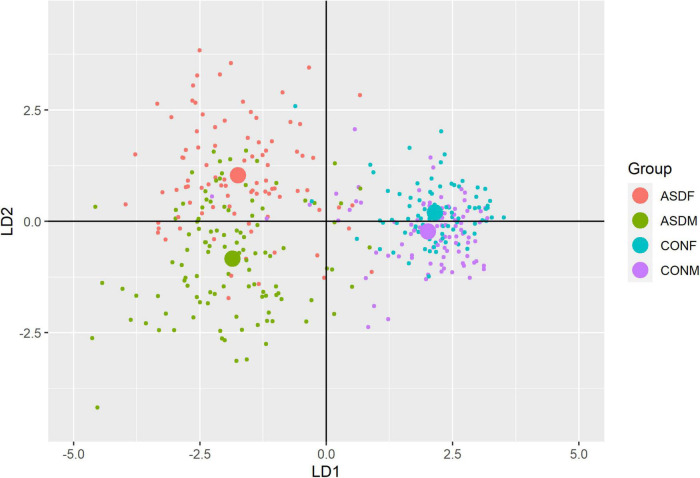
Linear discriminant axis 1 plotted against linear discriminant axis 2.

**FIGURE 2:**
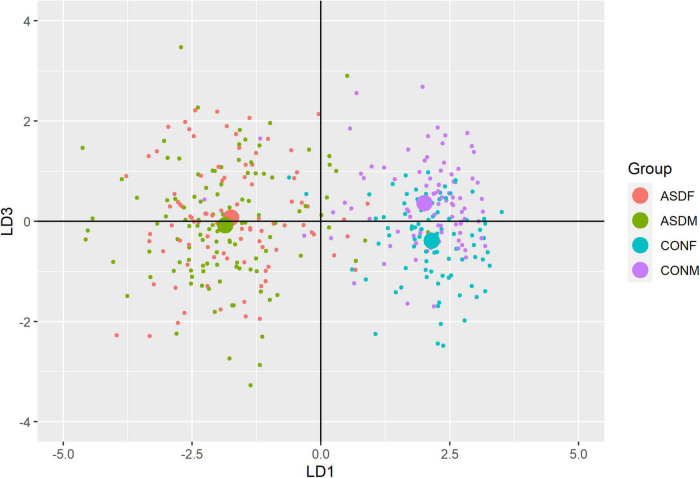
Linear discriminant axis 1 plotted against linear discriminant axis 3.

**FIGURE 3:**
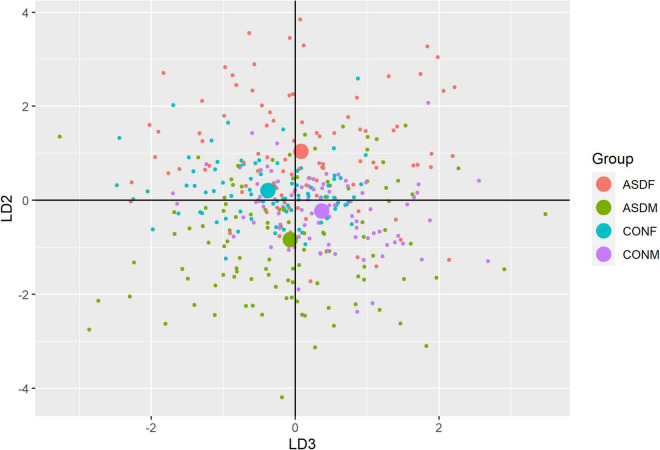
Linear discriminant axis 2 plotted against linear discriminant axis 3.

**FIGURE 4:**
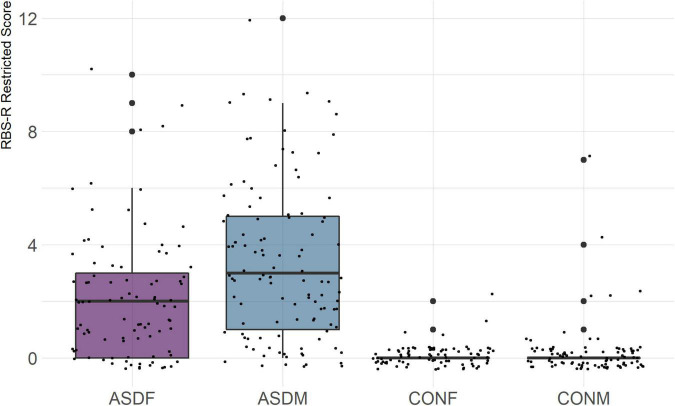
RBS-R Restricted Subscale.

**FIGURE 5:**
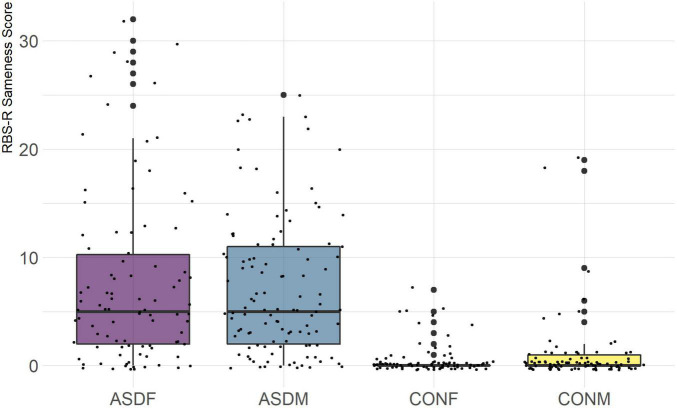
RBS-R Sameness Subscale.

**FIGURE 6:**
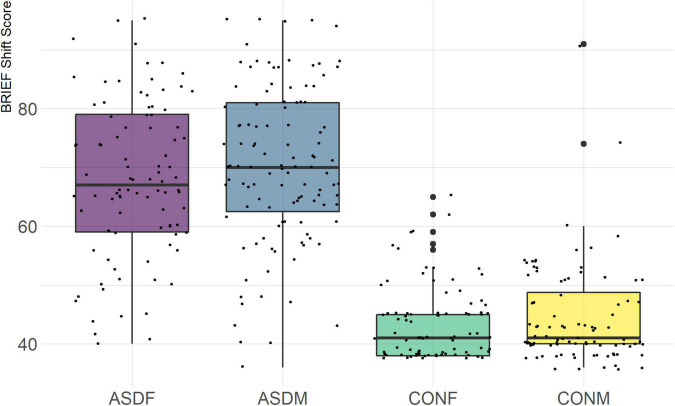
BRIEF Shift Subscale.

**FIGURE 7:**
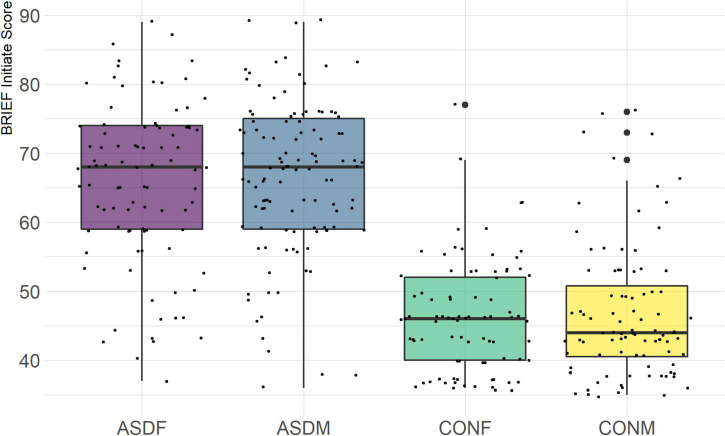
BRIEF Initiate Subscale.

**FIGURE 8:**
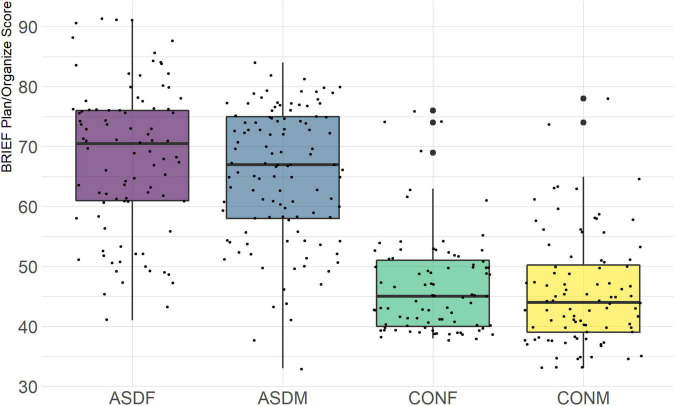
BRIEF Plan/Organize Subscale.

**FIGURE 9:**
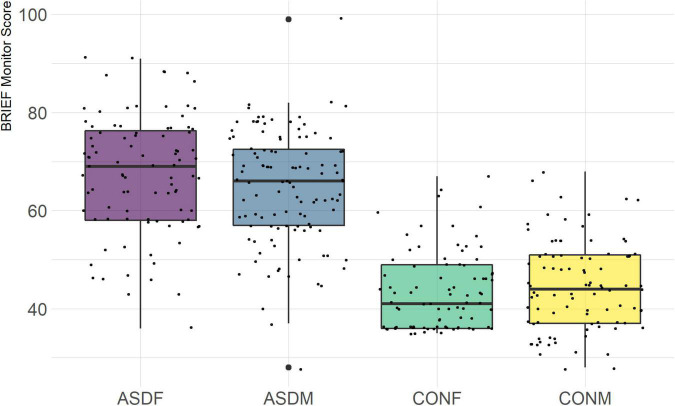
BRIEF Monitor Subscale.

**FIGURE 10:**
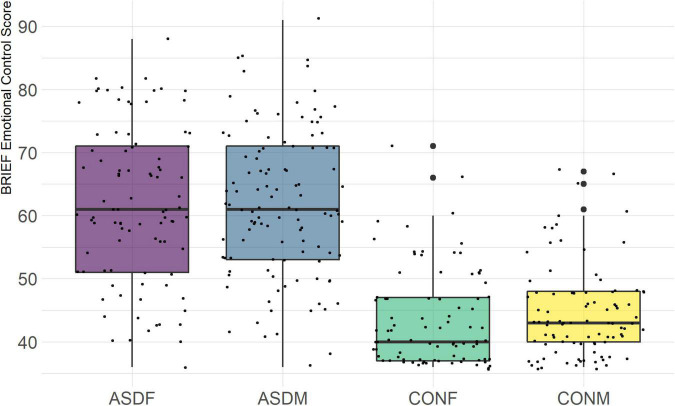
BRIEF Emotional Control Subscale.

**FIGURE 11:**
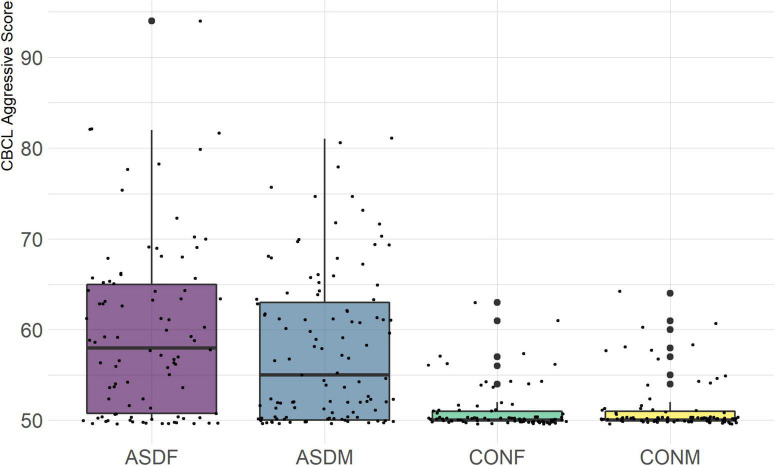
CBCL Aggressive Subscale.

**FIGURE 12:**
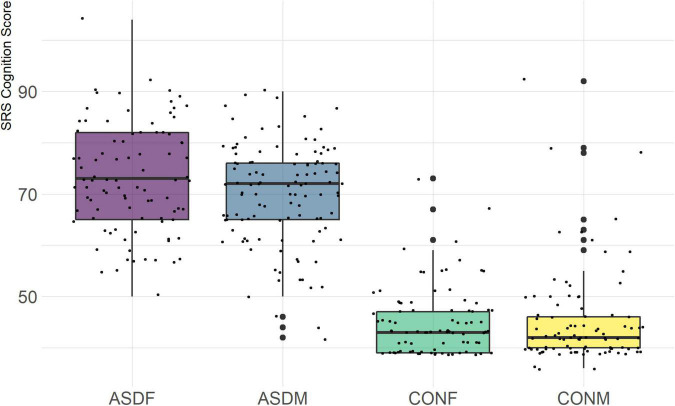
SRS Cognition Subscale.

Three distinct linear discriminant axes (LD1, LD2, and LD3) resulted from this analysis. LD1 explains 87.16% of the between-class variance, LD2 explains 11.25% of the between-class variance, and LD3 explains the remaining 1.58%. Note that whether a coefficient is positive or negative corresponds to the direction in which a measure weight “pulls” the different groups; for example, high scores (and therefore high dysfunction) in the CBCL Social Problems subdomain result in the overall distribution being split between the ASD group and the control group. This phenomenon is illustrated in Figures 1–3. The strongest discriminant coefficients for LD1 included the following measures in order of absolute value: CBCL Social Problems (−0.515), SRS Restricted/Repetitive Behavior (−0.478), Vineland Socialization (0.456), BRIEF Shift (−0.438), and CBCL Aggressive (0.430). The strongest discriminant coefficients for LD2 included the following measures in order of absolute value: RBSR Restricted Subscale (−1.113), BRIEF Shift (−0.974), SRS Cognition (0.772), BRIEF Initiate (−0.598), and BRIEF Plan/Organize (0.521). The strongest discriminant coefficients for LD3 included the following measures in order of absolute value: SRS Communication (−1.437), BRIEF Plan/Organize (−0.891), BRIEF Inhibit (0.839), Vineland Communication (−0.688), and SRS Restricted/Repetitive Behavior (0.664). A full accounting of these values can be found in Table 4. Cohort-wise comparisons of the raw values of the most relevant predictors can be found in Figures 4–12.

**TABLE 4:** Linear discriminant coefficients by measure for the linear discriminant analysis.

Measure name	LD1	LD2	LD3
BRIEF Emotional Control	−0.20297	**−0.39486**	0.05349
BRIEF Inhibit	0.19281	0.18188	0.83851
BRIEF Initiate	−0.03451	**−0.59808**	0.12022
BRIEF Monitor	−0.26000	**0.40408**	0.52330
BRIEF Organization Materials	0.34038	−0.00612	0.07616
BRIEF Plan/Organize	−0.21075	**0.52126**	−0.89132
BRIEF Shift	−0.43757	**−0.97355**	−0.26616
BRIEF Working Memory	−0.10153	−0.24019	−0.33680
CBCL Aggressive	0.42970	**0.44375**	−0.05251
CBCL Anxious	−0.02151	0.33984	0.26392
CBCL Attention	0.02869	0.16301	0.27304
CBCL Rulebreak	0.04911	−0.08144	−0.31350
CBCL Social Problems	−0.51466	−0.28194	−0.18579
CBCL Somatic Complaints	0.13999	0.01249	−0.18741
CBCL Thought	−0.11405	0.00873	−0.26736
CBCL Withdrawn	0.00195	0.06899	0.53452
CELF Formulate Sentences	0.28644	0.29849	0.19315
CELF Recall Sentences	−0.22209	0.21152	−0.48370
DAS Special Non-verbal	−0.33844	−0.19544	−0.23069
DAS Spatial Reasoning	0.14317	0.09762	0.32755
DAS Verbal Reasoning	0.07768	−0.18595	0.39518
RBS-R Compulsive	−0.01179	0.32377	0.17877
RBS-R Restricted	−0.15597	**−1.11277**	−0.34308
RBS-R Ritualistic	−0.03040	−0.07301	0.21968
RBS-R Sameness	0.39078	**0.40646**	0.04211
RBS-R Self-Injurious	−0.05025	−0.19514	0.08560
RBS-R Stereotyped	0.05553	0.09712	−0.08916
SRS Awareness	−0.11398	−0.17528	0.00108
SRS Cognition	−0.32790	**0.77152**	0.56659
SRS Communication	−0.32547	0.30805	−1.43705
SRS Motivation	0.21668	0.29158	−0.32940
SRS Restricted/Repetitive	−0.47764	0.26858	0.66440
Vineland Communication	0.16077	0.25030	−0.68800
Vineland Living Skills	−0.08184	0.30665	−0.31959
Vineland Socialization	0.45609	−0.22276	0.39264

Numbers highlighted in bold represent squared discriminant eigenvector coefficients greater than |0.15|.

A multivariate analysis of variance was conducted on the data to confirm the significance of the results of the LDA; the results of this analysis indicated highly significant results (Wilks’ lambda = 0.13065, *F(105, 1025)* = 9.501, *p* < 0.001). The individual analyses of variance for each linear discriminant axis were also significant (LD1: *F* = 684.4, *p* < 0.001; LD2: *F* = 172.0, *p* < 0.001; LD3: *F* = 32.08, *p* < 0.001). Figures of the linear discriminant axes plotted against each other can be found in Figures 1–3.

## Discussion

### Overview

The multivariate results of the present study indicate the diagnostic groups are linearly discernible by two key dimensions present in the phenotypic test battery: (1) a dimension separating groups by ASD diagnosis (LD1: 87% of variance explained) and (2) a dimension representing a diagnosis-by-sex interaction (LD2: 11% of variance explained). As evident in Figures 1, 2, there is a clear separation between the ASD and control group (LD1 axis), and there appears to be separation between the male and female ASD participants in Figure 1 (LD2 axis) but no such separation between male and female control participants. These results are confirmed in the class statistics table (Table 3), which displays sensitivity, specificity, and F-1 Score for each class. These are discussed in more detail below. A final dimension was not found to add to the ability to distinguish between the groups (LD3: 1.58% of variance explained) and so can be discounted. This provides compelling evidence for phenotypic differentiability between ASD males and ASD females.

### Advantages of a linear discriminant analysis

While any number of statistical models or machine learning approaches could have been adopted and applied in the analyses of the data included in this study, many suffer from a lack of omnibus tests of inferential statistical significance. Moreover, the contributions of individual variables to measuring between-group differences are often difficult to assess or even have access to in certain machine learning approaches. This is not to say that such methods are deficient or inappropriate; rather, probabilistic, non-linear, or other methods may not necessarily provide actionable information having clinical utility. In contrast, despite its multivariate nature, linear discriminant analysis provides a parsimonious and interpretable means for characterizing differences between groups, leveraging the covariance structure existing between sets of variables, which can be tested for statistical significance, and, finally, where the relative contributions of variables can be determined.

Thus, from a utility perspective, the linear discriminant analysis reported here likely has greater clinical applicability. Illustrating the sensitivity to a significant sex-by-diagnosis interaction may inform initial clinical assessment, identify those factors driving such differences, and which may be useful for customizing any therapeutic strategies specific to females suspected of an ASD diagnosis. Whereas machine learning and other modern approaches can help computers distinguish between groups, here we emphasize the potential for human interpretable analyses which optimize clinical utility.

### Classification metrics

The detailed class-wise statistics in Table 3 illustrate important context for this analysis. Sensitivity and precision both refers to the ability of the model to correctly identify members of a specific class but with different denominators: sensitivity is the true positives divided by the sum of true positives and false negatives, and precision is the true positives divided by the sum on true positives and false positives. The disparate sensitivity and precision values between the diagnostic groups reveal that, indeed, the linear discriminant model is highly accurate at distinguishing ASD from typically developing control participants. Also of note is the fact that the model was not as accurate differentiating the control males from control females as it was differentiating between ASD males and females, which indicates that these variables contribute only to the sex-wise differences in the ASD group. Additionally, ASD females were less likely to be correctly classified compared to ASD males, which likely reflects the male-tending bias of many of the phenotypic assessments used for assessing ASD.

### Contributing variables

The top discriminant variables in LD1 are the variables that exemplify the well-known differences between the ASD and neurotypical groups, such as difficulties with social interactions (CBCL social problems, Vineland Socialization), emotional control (CBCL Aggressive), and differences in effective communication (DAS Special Non-verbal, SRS Communication) (Elsabbagh and Johnson, 2007; Halladay et al., 2015). The difference in diagnostic group is apparent graphically in Figures 1, 2, the images that include LD1 as an axis.

### Sex-by-diagnosis interaction

A particularly relevant finding of this analysis exists with the strongest coefficients from LD2, since this is the axis along which the divide between ASD males and ASD females was the clearest. Specifically, these include several BRIEF-2 indices (Shift; Plan/Organize; Monitor; Emotional Control) as well as two RBS-R subdomains (Restricted; Sameness). The presence of these measures in the linear discriminant axis most implicated in ASD male-female separation suggests they may be important in the detection of ASD based on behavioral measures alone. Indeed, when a second, confirmatory linear discriminant analysis was run using only these predictors (squared LD2 coefficient > 0.15; highlighted in bold in Table 4), the discriminant axes and plots were comparable to the original analysis with the full array of predictors. They also may provide insight into the sex differences present in the ASD phenotype. Additional analysis is likely required to better determine how the BRIEF-2 and RBS-R subscales discriminate between the groups of interest. It is important to note that of these two measures, the BRIEF-2 is sex-normed and the RBS-R is not; this could have impacted the relative strength of the RBS-R subscales separating males from females, but it strengthens the results that suggest the BRIEF-2 is identifying latent traits specific to a sex-by-diagnosis disparity.

The overall strongest discriminator in the sex-by-diagnosis axis was the RBS-R Restricted Behaviors subscale. The Sameness subscale from the RBS-R assessment was also among the top discriminants in the sex-by-diagnosis axis. This is an interesting finding because previous research has indicated that repetitive behaviors can be subdivided into restrictive/repetitive sensory motor behaviors and insistence on sameness behaviors (Cuccaro et al., 2003; Bishop et al., 2013), which in other publications have been classified as low-order (restricted) and high-order (sameness) behaviors (Turner, 1999). Restricted behaviors can include dyskinesia, convulsions, and repeated manipulation of objects, while sameness behavior refers to a general insistence on routine consistency (Tian et al., 2022). The discriminant coefficients indicate these two subscales are representative of opposite group membership, where greater deficits in restricted behaviors are associated with ASD males and greater deficits in sameness behaviors are associated with ASD females.

Of the four BRIEF-2 subscales that most contributed to the ASD male-female discrimination, the Shift subscale was the strongest. This scale measures cognitive flexibility, or the ability to transition from one mental occupation to another (Gioia et al., 2000). The directionality of the Shift subscale coefficient in LD1 suggests that ASD participants exhibit far more dysfunction in this area than controls, which is corroborated by previous findings (Gioia et al., 2002; Blijd-Hoogewys et al., 2014). However, the directionality of this coefficient in LD2 suggests that ASD females exhibit less dysfunction in this area than ASD males (this finding contradicts earlier research, which suggests the opposite (White et al., 2017). The fact that this subscale was also a strong discriminator in the sex-by-diagnosis interaction axis may represent a clue as to the cause of the sex-based diagnostic disparity, as an inability to shift freely from activities or situations may be quite obvious to an observer. For example, some items on the BRIEF-2 that contribute to the Shift subscale include questions about being disturbed by a change of teacher or thinking too much about the same topic. Being able to mask this deficit could impact the decision to diagnose a child with ASD.

### Clinical implications

Males are diagnosed up to five times more frequently than females having ASD. Reasons posited for this effect involve the notion that females may require a greater environmental burden in order to cross the threshold normally seen in ASD males. Likewise, such a burden may have neurological and/or genomic determinants. Examination of neuropsychological and behavioral assessments via detailed multivariate analysis illustrated that while typically developing males and females are indistinguishable, males and females diagnosed with ASD were clearly separable. Assessments maximally contributing to this sex-by-diagnosis interaction were reflective of executive function, cognitive, and emotional control as well as restricted behaviors. This suggests that, of the broad range of assessments included in the presence analysis, the BRIEF-2 and RBS-R may be particularly sensitive to these sex-driven differences. Neuropsychologists utilizing sub-scales of these metrics, in particular, may be able to better fine-tune options for clinical therapeutic strategies specific to females suspected of being on the Autism spectrum.

### Future directions

The multimodal richness inherent in this dataset would be enhanced through the inclusion of neuroimaging and genomic data. Doing so would provide further evidence for a sex-based differences in the ASD phenotype as it relates to neurological and genomic contributions. Previous research has indicated strong evidence for genetic differences between ASD male and ASD females (Jack et al., 2021), which lends credence to the female protective effect hypothesis and may provide another avenue to analyze these data. Additional and intensive classification analyses, such as bagged random forests and support vector machines, focused on more targeted phenotypic variables could also help to refine the results of this preliminary analysis. Evidence from the ASD literature suggesting multimodal diagnosis methods can be more effective than the gold-standard survey methods by including such neurological tools as EEG biomarkers though these techniques are still being developed and are not sufficiently robust for definitive diagnosis (Tanu, and Kakkar, 2019). While measures employed here were able to be classified effectively with a parametric model is encouraging for future multimodal analyses on neuropsychological assessments, non-parametric methods could be advantageous for successful classification using time-dependent data. Indeed, deep learning techniques deployed on EEG signal for ASD classification in other studies have achieved high accuracy and represent path toward automated ASD diagnosis (Wadhera et al., 2021), although the interpretability of deep learning models remains limited. Finally, this sample consists of highly verbal, average IQ ASD participants and most measures are parent-reported, which could have impacted the generalizability of the results. Using gender identity in addition to biological sex is another potential future direction, and our continued research has been diligent about collecting this information.

## Final conclusion

A phenotypic battery of neuropsychological and behavioral assessments subjected to multivariate linear discriminant analysis revealed diagnosis, as well as sex-by-diagnosis related dimensions which distinguished ASD from typically developing control participants. Main drivers of the latter were sub-scales of the BRIEF-2 and RBS-R, both of which are measures pertaining to contextual behavior. These phenotypic assessments, in particular, may reflect useful means by which to tailor therapeutic interventions and clinical approaches specifically aimed at addressing ASD in females.

## The GENDAAR Consortium members

Katy Ankenman, Sarah Corrigan, Dianna Depedro-Mercier, Nadine Gaab, Desiree Guilford, Abha R. Gupta, Shafali Jeste, Cara M. Keifer, Anna Kresse, Erin Libsack, Jennifer K. Lowe, Erin MacDonnell, Nicole McDonald, Adam Naples, Charles A. Nelson, Emily Neuhaus, Pamela Ventola, Olivia Welker, and Julie Wolf.

## Data availability statement

The datasets presented in this study can be found in online repositories. The names of the repository/repositories and accession number(s) can be found below: https://nda.nih.gov/edit_collection.html?id~(~=2021.

## Author contributions

AJ, CS, EA, SB, MD, Nadine Gaab, JDVH, RB, Charles Nelson, SW, Abha Gupta, and KP: conceptualization. AJ, CS, EA, SB, MD, Nadine Gaab, JDVH, Abha Gupta, and KP: methodology. AJ, CS, JDVH, JE, ZJ, and CT: software. AJ and CS: formal analysis, writing—original draft, and visualization. AJ: investigation. JDVH and KP: resources. JDVH, JE, ZJ, and CT: data curation. AJ, Abha Gupta, and KP: writing—review and editing. EA, SB, MD, Nicole McDonald, JDVH, RB, DG, JM, Nicole McDonald, SW, Abha Gupta, and KP: supervision. EA, SB, MD, Nicole McDonald, JDVH, RB, DG, JM, Charles Nelson, SW, and KP: project administration. KP: funding acquisition. All authors contributed to the article and approved the submitted version.
